# Crystal structure of Qa-1^a^ with bound Qa-1 determinant modifier peptide

**DOI:** 10.1371/journal.pone.0182296

**Published:** 2017-08-02

**Authors:** Ge Ying, Jing Wang, Vipin Kumar, Dirk M. Zajonc

**Affiliations:** 1 Division of Cell Biology, La Jolla Institute for Allergy and Immunology (LJI), La Jolla, California, United States of America; 2 Department of Medicine, University of California San Diego, La Jolla, California, United States of America; 3 Department of Internal Medicine, Faculty of Medicine and Health Sciences, Ghent University, Ghent, Belgium; University College London, UNITED KINGDOM

## Abstract

Qa-1 is a non-classical Major Histocompatibility (MHC) class I molecule that generally presents hydrophobic peptides including Qdm derived from the leader sequence of classical MHC I molecules for immune surveillance by NK cells. Qa-1 bound peptides derived from the TCR Vβ8.2 of activated T cells also activates CD8^+^ regulatory T cells to control autoimmunity and maintain self-tolerance. Four allotypes of Qa-1 (Qa-1^a-d^) are expressed that are highly conserved in sequence but have several variations that could affect peptide binding to Qa-1 or TCR recognition. Here, we determined the structure of Qa-1^a^ with bound Qdm peptide. While the overall structure is very similar to that of Qa-1^b^, there are several amino acid differences around the peptide binding platform that could affect TCR recognition. Most notably, two amino acid substitutions are found in the pocket P2, which binds the anchor residue Met2 of the Qdm peptide. These residues affect both the size and shape of the binding pocket, as well as affect the charge at physiologic pH, suggesting Qa-1^a^ and Qa-1^b^ could present slightly distinct peptide reservoirs, which could presumably be recognized by different populations of CD8^+^ T cells.

## Introduction

Peptide presentation by Major Histocompatibility (MHC) proteins is important for initiating adaptive immune responses and immune surveillance by T cells ad NK cells [1, 2]. While classical MHC peptide-antigen presenting molecules are highly polymorphic and either activate cytotoxic CD8^+^ T cells (MHC class I) or CD4^+^ T helper cells (MHC class II), several non-classical MHC I-like (MHC Ib) molecules exist, that exhibit limited polymorphism [3, 4]. Of these MHC Ib molecules, murine Qa-1 consists of the 4 allotypes Qa-1^a^, -1^b^, -1^c^, and 1^d^ [5], while humans encode the ortholog HLA-E [6, 7]. In addition to antigen recognition by T cells, Qa-1 or HLA-E are recognized by NK cells, most notably through the binding by the inhibitory heterodimeric receptor CD94/NKG2A [8, 9]. Qa-1 has been shown to present the nonamer leader peptide (AMAPRTLLL) found in most classical MHC Class I alleles of H-2D and H-2L [10]. Loading of this leader peptide, called Qa-1 determinant modifier (Qdm) into Qa-1 is dependent on the transporter associated with antigen processing (TAP) [10, 11]. Both Qa-1 and HLA-E monitor the steady-state expression of MHC Ia molecules and relay that information to the cell surface where they engage the inhibitory NK receptor CD94/NKG2A to prevent killing of a healthy antigen presenting cell [8, 9]. Several viruses and tumors acquired the ability to downregulate MHC I expression to evade immune surveillance by T cells [12, 13]. However, the reduced availability of the MHC I leader peptides diminish cell-surface expression of Qa-1a and HLA-E molecules and a lack of inhibitory NK cell signaling. In addition NK cell activating ligands upregulated upon viral infection, lead to NK cell activation and the killing of the infected antigen-presenting cell [14].

The interaction of CD94/NKG2A with HLA-E, and Qa-1 is both specific for the Qa-1antigen-presenting molecule and the Qdm peptide. A closely related paralog of Qa-1, encoded by the gene H2-T11 can associate with the Qdm peptide but does not bind CD94/NKG2A [15]. Furthermore, CD94 and NKG2A each form contacts with several upward facing Qdm residues [16–19].

In addition to Qdm, Qa-1 molecules have been shown to associate with many more peptides that are derived from both endogenous or exogenous proteins. The source proteins of these peptides include heat shock protein 60, preproinsulin, TCR Vβ chain, *Salmonella typhimurium* GroEL, and an unidentified protein from *Listeria monocytogenes* [7, 20–23].

Recently, Qa-1-restricted CD8^+^ regulatory T (Treg) cells have been identified and their role in maintaining self-tolerance has been established [24]. A population of these Tregs are Foxp3-negative and express the CD8αα homodimer [23]. They present a peptide derived from the TCR Vβ8.2 chain, kill activated but not naïve Vβ8.2 T cells leading to control of experimental autoimmune encephalomyelitis (EAE) [23, 25].

Since Qa-1 expresses four allotypes that are highly conserved in sequence, we determined the crystal structure of Qa-1^a^ bound to Qdm to address the question, whether differences in the binding and presentation of Qdm between Qa-1^a^ and Qa-1^b^ exist that could potentially affect TCR specificity among different CD8^+^ T cell populations.

## Materials and methods

### Gene constructs and expression cloning

Synthetic DNA encoding the mouse Qa-1^a^ extracellular domain (amino acids 21–297) was obtained from GenScript, as a codon optimized construct for expression in *E*.*coli*. We further incorporated a 5’ NdeI site and a 3’-XhoI site for subsequent subcloning into the expression vector pET-22b (NdeI-XhoI). A stop-codon was introduced just before the XhoI site of the Qa-1^a^ construct to prevent expression of the vector containing C-terminal his-tag. The use of the NdeI site of pET22b+ also resulted in the removal of the pelB leader from the expressed construct, since we aimed to express the protein in inclusion bodies for refolding. The mouse *β*-2 microglobulin (*β*2m) cDNA (residues 21–119) was cloned into pET-22b using the same restriction sites. Both expression plasmids were separately transformed into *E*.*coli* BL21 (DE3) cells. All constructs were verified by sequencing.

### Protein expression and inclusion body purification

*E*.*coli* BL21 (DE3) cells (New England Biolabs) were grown in TB media until D_600_ of 0.9 and recombinant protein expression was induced with 1mM IPTG for 4hr at 37°C. Both proteins were expressed in insoluble inclusion bodies. *E*.*coli* cells were harvested by centrifugation (5000g for 15minutes at 4°C), resuspended in the ice-cold lysis buffer (100mM Tris-HCl pH 7.0, 5mM EDTA, 5mM DTT, 0.5mM PMSF) and lysed by passing through a microfluidizer under 20K psi (Microfluidics). Inclusion bodies were sedimented (50,000g for 20minutes at 4°C) and resuspended using wash buffer (100mM Tris-HCl pH 7.0, 5mM EDTA, 5mM DTT, 2M Urea, 2% w/v Triton X-100). This step was repeated twice and detergent was removed by resuspending inclusion bodies in detergent-free wash buffer (100mM Tris-HCl pH 7.0, 5mM EDTA, 2mM DTT). Finally, the purified inclusion bodies were denatured in 50mM Tris-HCl pH 7.0, 5mM EDTA, 2mM DTT, 6M Guanidine-HCl, protein purity assessed by SDS-PAGE, and quantitated by Bradford assay.

### Refolding and protein purification

The Qa-1^a^/β2m/Qdm trimeric complex was formed by refolding of denatured inclusion body proteins. Briefly, 4mg of Qdm nonapeptide (AMAPRTLLL from GenScript, >95% purity, 10mg/ml in DMSO), followed by the mixture of 2.75mg of denatured Qa-1^a^ and 3mg of denatured β2m, were added dropwise into 250ml of high-speed stirred cold refolding buffer (100mM Tris-HCl pH 8.0, 2mM EDTA, 400mM L-arginine, 5mM reduced glutathione, 0.5mM oxidized glutathione, 0.2mM PMSF) and then incubated at 4°C with medium-speed stirring. Mixture of 2.75mg of Qa-1^a^ and 3mg of β2m without Qdm peptide was added twice after every 12 hours. The final molecular ratio of heavy chain: light chain: peptide was around 1:3:16. After 3 days stirring at 4°C, the refolding mixture was filtered through a 0.22μm membrane and concentrated to 0.5ml (Millipore Amicon 30K centrifugal filter) for size exclusion chromatography (SEC, Superdex S200 10/300) in 10mM Tris-HCl pH 8.0, 150mM NaCl. Fractions containing refolded Qa-1^a^/β2m/Qdm trimeric complex were analyzed by SDS-PAGE, and then pooled and concentrated for subsequent crystallization. Successful incorporation of Qdm peptide was confirmed by MALDI-TOF (The Scripps Research Institute).

### Crystallization and data collection

The refolded Qa-1^a^/β2m/Qdm trimeric complex was buffer exchanged into 10mM Tris-HCl pH 8.0, 5mM NaCl at 4.5mg/ml and a crystallization screen was set up in a wide range of conditions at 22°C using the sitting drop vapor diffusion method. The initial Qa-1^a^/β2m/Qdm crystals were grown as a shower of needles in 100mM sodium acetate pH4.5, 5% (w/v) PEG1000, 50% ethylene glycol. The condition was optimized for both buffer pH value and precipitant concentration for growth of better diffracting crystals. The diffraction-quality rod-like crystals were finally obtained in 100mM HEPES pH6.5, 5% (w/v) PEG1000, 40% ethylene glycol and flash cooled in liquid nitrogen. Diffraction data for Qa-1^a^/β2m/Qdm crystal were collected remotely at beam line 7–1 at the Stanford Synchrotron Radiation Light source (SSRL).

### Structure determination and refinement

Diffraction data for Qa-1^a^/β2m/Qdm crystal were processed and scaled using HKL2000. The Qa-1^a^/β2m/Qdm structure was solved by molecular replacement method using the protein coordinates from structure of Qa-1^b^/β2m/Qdm (PDB ID 3VJ6, with Qdm peptide removed) using PHASER [26] as part of the CCP4 suite [27]. The model was built and refined iteratively using COOT [28] and REFMAC5 [29], and monitored by a continuous drop in *R*_*free*_ values and improvement in electron density. Refinement was carried out to a final R_cryst_ and R_free_ of 21.8% and 24.5%. Data collection and refinement statistics are presented in Table 1.

**Table 1:** Data refinement and statistics.

Data collection statistic	Qa-1^a^/Qdm
PDB ID	5VCL
Space group	P2_1_22_1_
Cell dimension	
*a*, *b*, *c*, (Å)	67.2, 75.9, 105.2
α, β, γ (°)	90, 90, 90
Resolution range (Å) [outer shell]	40.0–2.04 [2.11–2.04]
No. of unique reflections	34,634 [3,228]
R_meas_ (%)	11.2 [67.0]
R_pim_ (%)	4.9 [29.8]
Multiplicity	4.9 [4.5]
Average I/σI	16.3 [2.1]
Completeness (%)	98.8 [93.3]
**Refinement statistics**	
No. atoms	3,309
Protein	3,091
Peptide	68
Water	120
Glycerol/sodium	24/6
Ramachandran plot (%)	
Favored	97.4
Allowed	99.7
R.m.s. deviations	
Bonds (Å)	0.008
Angles (°)	1.31
B-factors (Å^2^)	
Protein	38.4
Peptide	33.7
Water	41.2
Glycerol/sodium	57.0/45.6
R factor (%)	21.7
R_free_ (%)	24.6

### Structural analyses and presentation

The quality of the models were examined using Molprobity [30].

Buried surface areas (BSA) and atomic contacts were calculated using PDBePISA (http://www.ebi.ac.uk/msd-srv/prot_int/pistart.html). Figures were prepared using PyMOL (Schroedinger).

### Accession numbers

The coordinates and structure factors for Qa-1^a^ have been deposited in the Protein Data Bank (www.rcsb.org) with codes 5VCL.

## Results

### Qa-1^a^/Qdm complex preparation

**Fig 1:**
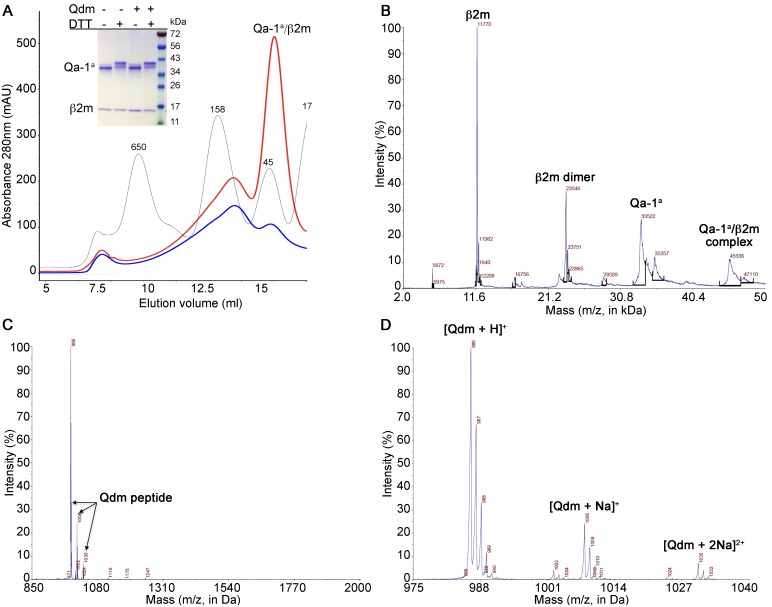
Qa-1^a^-Qdm complex preparation and analysis. (A) Size exclusion chromatography of Qa-1^a^ refolded in the presence (red) or absence (blue) of Qdm peptide. Molecular weight standard as grey line with MW in kDa. Insert shows SDS Page of the pooled 45 kDa peak fractions under reducing (+DTT) and non-reducing (-DTT) conditions, refolded with (+) or without (-) Qdm peptide. (B) MALDI-TOF of the Qa-1^a^/Qdm complex. (C, D). MALDI-TOF analysis identified bound Qdm peptide.

Qa-1^a^ was expressed and refolded together with β2m and the Qdm peptide AMAPRTLLL. Successful refolding and complex formation was detected by size exclusion chromatography on a Superdex S200 column and identification of a peak corresponding to a protein of a molecular weight of 45 kDa (Fig 1). Pooled fractions of that peak were subjected to SDS PAGE and contained both Qa-1^a^ heavy chain, non-covalently bound toβ2m. Proper intramolecular disulfide bond formation in Qa-1^a^ was indicated by a different mobility of the Qa-1^a^ band between non-reducing and reducing SDS-PAGE. Interestingly, Qa-1 ^a^ can also refold, albeit with lower efficiency, without addition of a peptide ligand, since a soluble 45 kDa Qa-1^a^/Qdm complex was obtained that showed the same intramolecular disulfide bond formation compared to refolding with the Qdm peptide (Fig 1A). This is in stark contrast to classical MHC I molecules, which only form stable complexes in the presence of a peptide ligand. Successful incorporation of the Qdm peptide was determined by MALDI-TOF (Fig 1B–1D). The Qdm peptide has a molecular weight of 985.2 Da and a dominant peak corresponding to a MW of 986 was identified, with two minor peaks (MW of 1008 Da and 1030 Da) indicating the presence of protonated Qdm, as well as sodium adducts of the peptide (Fig 1C and 1D).

### Qa-1a/Qdm crystal structure

**Fig 2:**
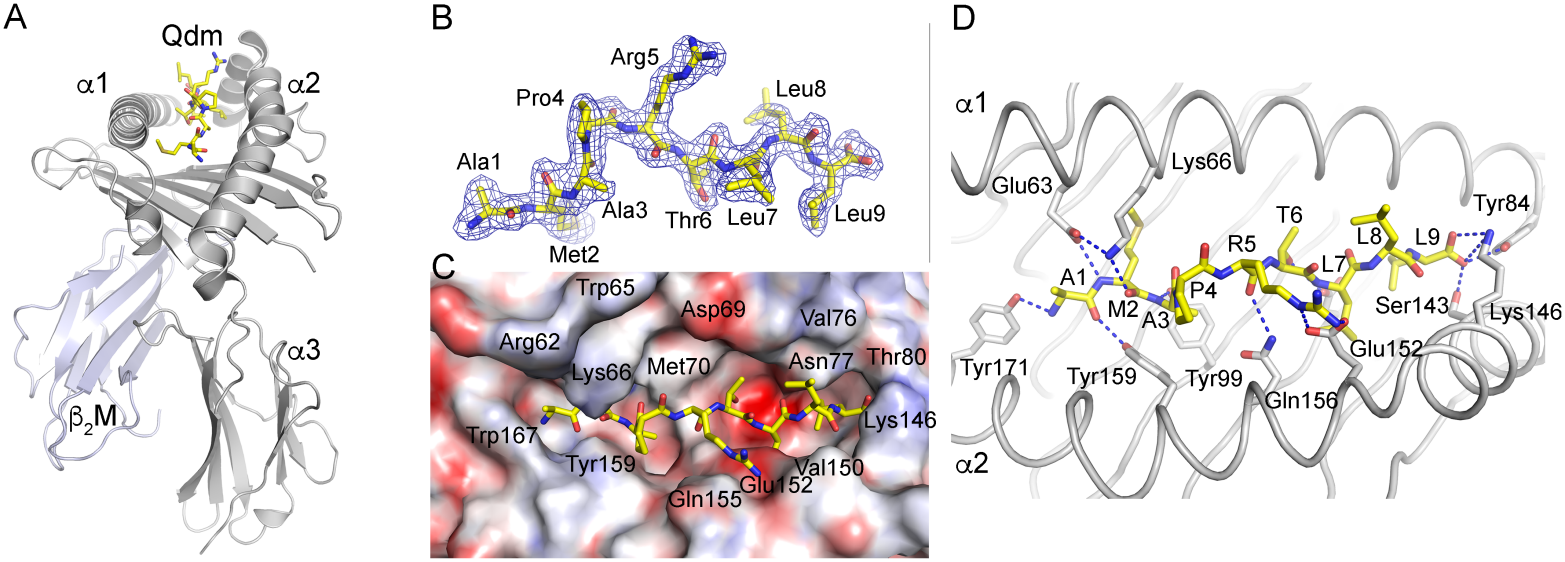
Structure of the Qa-1^a^-Qdm complex. (A) Cartoon representation with Qa-1^a^ heavy chain in grey, β_2_m in blue-grey and Qdm as yellow sticks. (B) 2Fo-Fc electron density mesh contoured at 1σ around the Qdm peptide. (C) Top view, looking down onto the Qdm binding site with Qa-1^a^ colored by electrostatic potential (from -30 to +30 kT/e). Red, negative and blue, positive charge. (D) Hydrogen-bond interactions between Qa-1^a^ and Qdm peptide indicated as blue dotted lines.

The Qa-1^a^/Qdm complex was crystallized in space group P21 2 21 with one complex per asymmetric unit, and diffraction data was collected to 2.04 Å resolution (Table 1). The structure was determined by molecular replacement using the coordinates of Qa-1^b^ as the search model [31]. The structure was refined to final R_cryst_ and R_free_ values of 21.8% and 24.5%, respectively. The overall structure of Qa-1^a^ follows that of other MHC I molecules. The Qa-1^a^ heavy chain forms three domains, α1, α2, and α3, with α1-α2 forming the antigen binding groove lined by the two anti-parallel α-helices α1 and α2, which sit above an anti-parallel β-sheet platform. The α3 domains forms a typical Ig-fold, binds non-covalently to the Ig superfamily member β2-microglobulin (β2m) and together both β2m and α3 domain support the antigen binding platform (Fig 2A).

### Qa-1^a^/Qdm peptide interaction

The Qdm peptide AMAPRTLLL binds in an elongated fashion tightly to the binding groove, as indicated by very well-defined electron density over the entire length of the peptide (Fig 2B and 2C). The peptide is bound via 14 H-bonds, 3 salt bridges, 3 water-mediated hydrogen bonds and 1 sodium-mediated hydrogen bond (Table 2). The presence of a proline residue results in a ‘kink’ in the peptide, and together with the underlying Tyr99 residue of Qa-1^a^ leads to a slight separation of the peptide binding groove into two halves. The N-terminal half is slightly smaller and mostly hydrophobic in nature, while the C-terminal half is slightly larger and contains a pronounced negative charge due to the presence of Glu116 protruding from the β-sheet platform. The smaller N-terminal pocket also contains a negatively charged residue Glu63, however the presence of neighboring Lys66 partially neutralizes this charge (Fig 2C). Surprisingly, however the N-terminus of the peptide, especially Ala1 and Met2 are bound mostly through hydrogen-bond interactions with backbone oxygens and amide, while the C-terminal part of the peptide that is accommodated in the negatively charged area of the peptide-binding groove is bound mostly through vdW interactions via Leu7 and Leu9. Here, hydrogen bond interactions are restricted to the backbone oxygen of Arg5 with Qa-1^a^ residue Gln156, as well as the carboxyl terminus of Leu9 with Qa-1^a^ groove-lining residues Tyr84, Ser143, and Lys146.

**Table 2:** D8 epitope residues and buried surface area.

Peptide Residue	Qa-1^a^ Residue	Interaction^a^
*Ala*^*1*^		
Ala	Tyr^7^, Glu^63^, Tyr^159^, Tyr^167^, Tyr^171^,	vdW
Ala^N^	Tyr^7 OH^, Tyr^171 OH^	H-bond
Ala^O^	Tyr^159 OH^	H-bond
*Met*^*2*^		
Met	Tyr^7^, **His**^**9**^, Met^45^, Glu^63^, Lys^66^, **Ala**^**67**^, Tyr^99^, Tyr^159^	vdW
Met^N^	Glu^63 OE2^	H-bond
Met^O^	Lys^66 NZ^	H-bond
*Ala*^*3*^		
Ala	**Lys**^**66**^, Met^70^, **Trp**^**97**^, Tyr^99^, Tyr^159^	vdW
Ala^N^	Tyr^99 OH^	H-bond
*Pro*^*4*^		
Pro	Tyr^159^	vdW
*Arg*^*5*^		
Arg	Trp^97^, Val^150^, Glu^152^, Gln^155^, Gln^156^,	vdW
Arg^NE^	Glu^152 OE2^	Salt bridge
Arg^NH2^	Glu^152 OE1^	Salt bridge
Arg^NH2^	Val^150 O^	H-bond
Arg^O^	Gln^156 NE2^	H-bond
*Thr*^*6*^		
Thr	Met^70^, Asn^73^, Phe^74^, Trp^97^, Glu^116^	vdW
Thr^OG1^	Glu^116^	Sodium-mediated H-bond
*Leu*^*7*^		
Leu	Asn^77^, Glu^116^, Ile^124^, Trp^133^, Ser^147^	vdW
Leu^O^	Asn^77 OD1^	H-bond
*Leu*^*8*^		
Leu	Asn^77^, Lys^146^	vdW
Leu^N^	Ser^147 OG^, Glu^152 OE1^	Water-mediated
Leu^O^	Ser^143 O^, Ser^147 OG^	H-bonds
*Leu*^*9*^		
Leu	Asn^77^, Thr^80^, Tyr^84^, Leu^95^, Glu^116^, Ser^143^, Lys^146^	vdW
Leu^N^	Asn^77 ND2^	H-bond
Leu^O^	Ser^143 OG^, Tyr^84 OH^	H-bond
Leu^OXT^	Lys^146 NZ^	Salt bridge
Leu^OXT^	Thr^80 OG1^	Water-mediated H-bond

^a^Atomic contacts where calculated using CONTACT as part of CCP4 [27] and PISA (http://www.ebi.ac.uk/msd-srv/prot_int/pistart.html)using the following distances. Van der Waals (vdW) interactions <4 Å, H-bonds <3.5 Å, and salt bridges <4.5 Å.

### Comparison between Qa-1^a^ and Qa-1^b^

**Fig 3:**
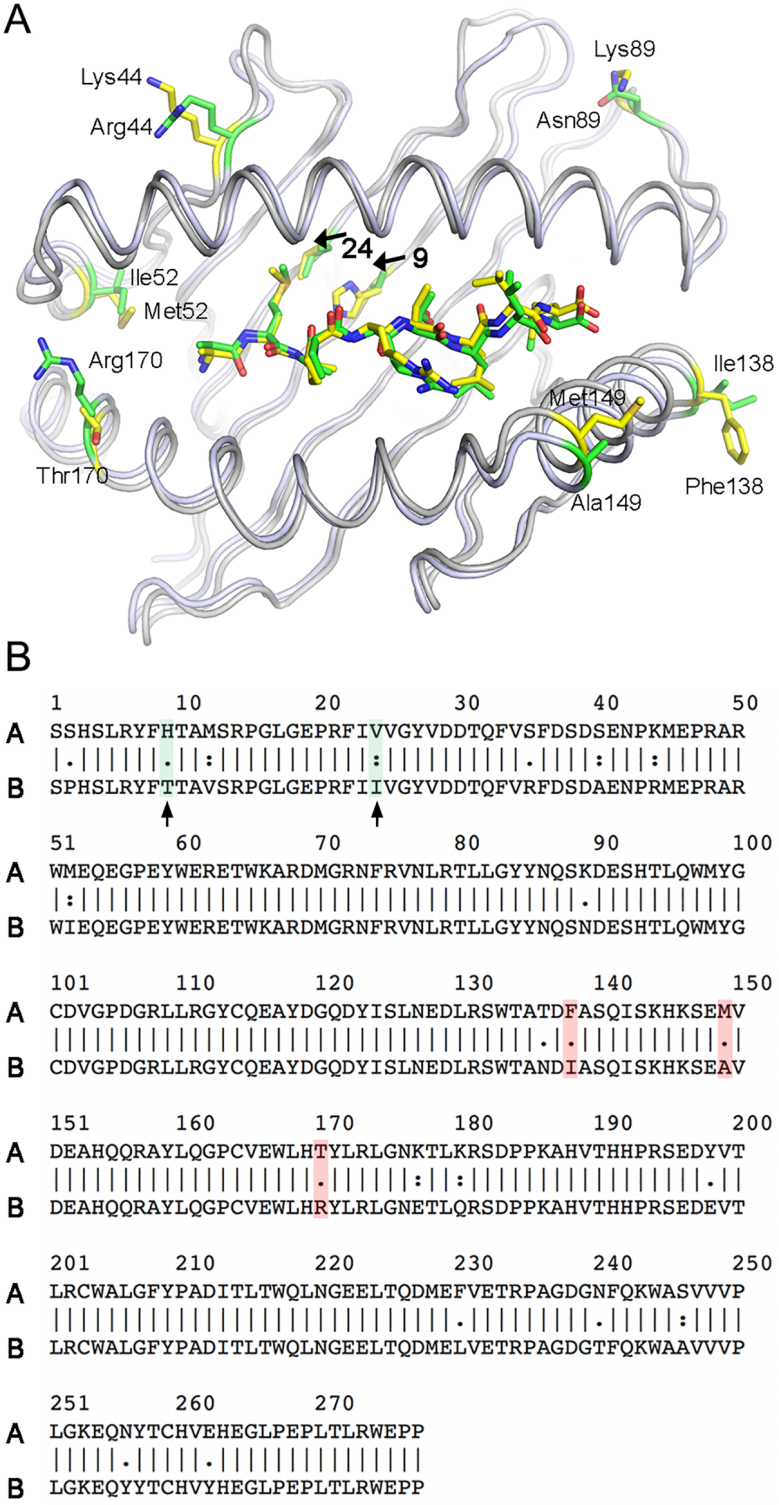
Comparison between Qa-1^a^ and Qa-1^b^. (A) Superimposition of Qa-1^a^ heavy chain with Qa-1^b^. Qdm peptides and several residues that are not conserved between both isoforms are shown as yellow (Qa-1^a^) and green (Qa-1^b^) sticks, while the Qa-1 proteins are shown as grey ribbons. Arrows in upper panel indicate amino acid differences that affect the peptide binding pocket of Qa-1^a^. (B) Sequence alignment between Qa-1^a^ (A) and Qa-1^b^ (B). Green shaded residues correspond to amino acid differences in the peptide binding pocket (P2) and are marked by an arrow, while red shaded residues are exposed on the α2-helix and could be contacted by NK cell or T cell receptors. Other amino acid differences that are located away from the antigen-binding groove are not highlighted in A, with the exception of residue 52, which lies underneath the α1-helix.

Qa-1^a^ is 92.4% identical in protein sequence to Qa-1^b^, for which the crystal structure is also available [31] (Fig 3). Over the entire ectodomain of the Qa-1^a^ heavy chain (277 amino acids), 15 amino acid variations are located in the α1-α2 domain and 6 variations are located in the α3 domain. Within the α1-α2 domain, three amino acids helix (Phe138, Met149, and Thr170) are upward facing along the α2- helix, while residues Lys44 and Lys 89 are located at loops of the β-sheet platform next to the α1-helix. These residues are in positions, where they could affect both binding to specific TCRs or NK receptors, such as CD94/NKG2A. Especially Lys89 and Thr170 are equivalent positions of CD94/NKG2A contact residues of the human ortholog HLA-E. Since these residues differ between Qa-1^a^ and Qa-1^b^ they could likely result in different binding affinities toward CD94/NKG2A. In HLA-E, both residues form either a salt-bridge (Arg170 of HLA-E) or a H-bond (Glu89 of HLA-E) with residues of the CD94 subunit of the CD94/NKG2A heterodimer. Most other amino acids that differ between Qa-1^a^ and Qa-1^b^ are not close to the receptor binding site and are, therefore, not highlighted in Fig 3.

### Differences in the Qa-1 binding pocket P2

**Fig 4:**
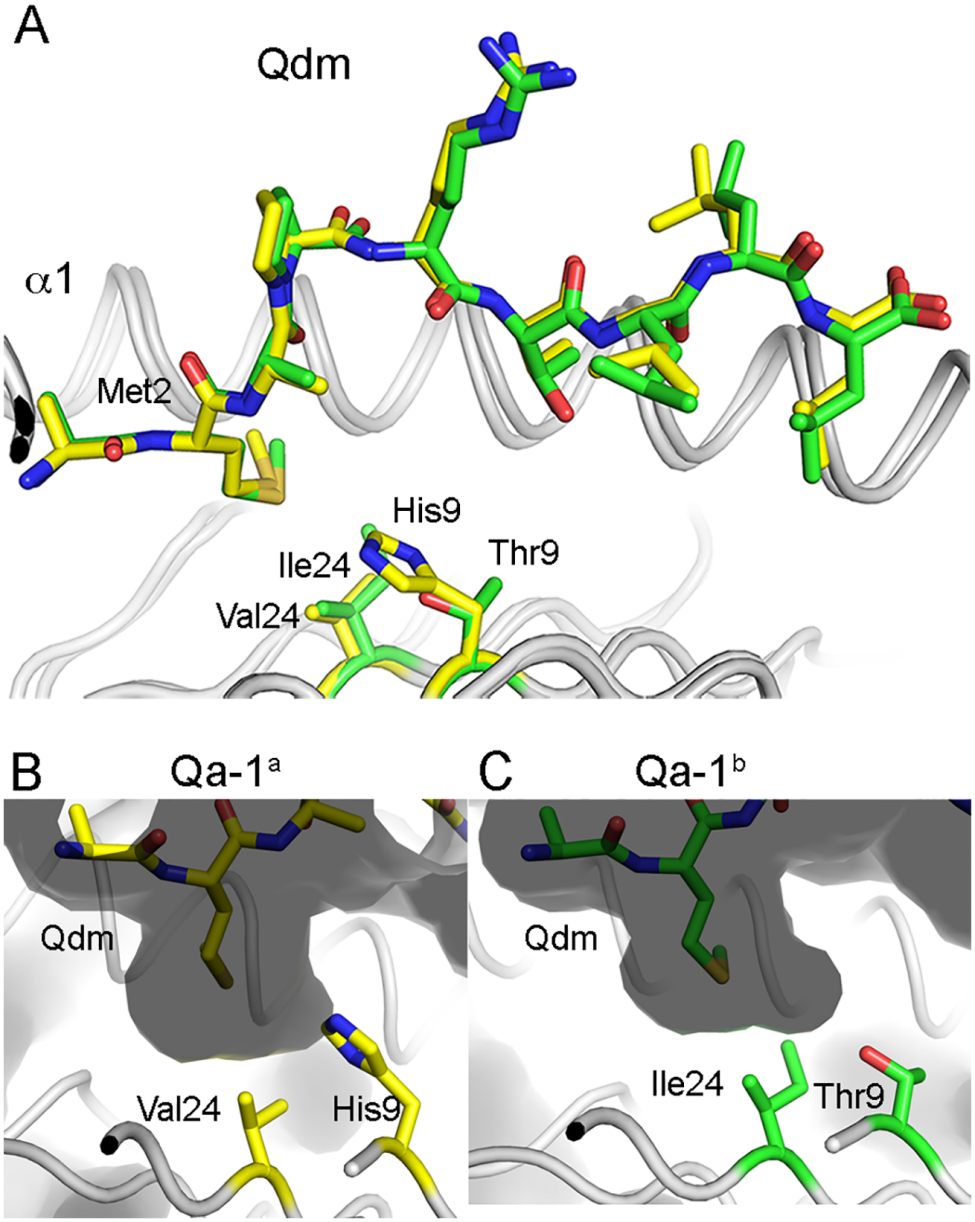
Difference in the peptide binding pocket between Qa-1^a^ and Qa-1^b^. (A) Presentation of the Qdm peptide by Qa-1^a^ (yellow) and Qa-1^b^ (green). (B) Qa-1^a^ binding pocket P2 shown as a molecular surface with the two non-conserved Qa-1^a^ residues shown as stick in yellow. (C) Binding pocket P2 of Qa-1^b^ with residues colored green.

While the Qdm peptide binding to both Qa-1^a^ and Qa-1^b^ appears almost identical, except for slight differences in the position of Leu7 and Leu8, two amino acid variations are located inside the peptide binding groove (His9 and Val24). These two resiudes together could affect both shape and charge of the binding pocket P2, which is an anchor pocket for Qdm residue Met 2 (Fig 4). While threonine is uncharged at neutral pH (pH 7.0), histidine is positively charged. As such, both shape and charge differences between Qa-1^a^ and Qa-1^b^ could result in differences in the peptide-binding specificities between both Qa-1 isotypes and as such affect T cell and NK cell receptor recognition and possibly function.

## Discussion

We report the crystal structure of the non-classical MHC–Ib allele Qa-1^a^ in complex with the Qdm peptide. Generally non-classical MHC-Ib molecules, such as Qa-1 or the human ortholog HLA-E exhibit limited polymorphism and present the leader sequences of classical MHC-Ia molecules to NK cells for recognition by the inhibitory heterodimeric receptor NKG2A/CD94. However, several studies have identified a surprisingly large panel of peptides that can be presented by Qa-1 or HLA-E [32–34]. While some of these peptides have both anchor residues Met at P2 and Leu at P9, as found in the high affinity Qdm peptide, several others do not share any sequence similarities with Qdm, suggesting a different mode of binding to Qa-1 or HLA-E [35]. An alternate binding mode is supported by Qa-1^b^ binding studies with Qdm peptides in which each amino acid was replaced with lysine. Met2 and Leu9 where crucial for binding since exchange against lysine led to a 1000-fold increase in IC_50_ [18]. In addition, a Qa-1^a^ nonamer peptide ligand (p42-50) has been identified that lacks the crucial Leu9 amino acid, suggesting that not all Qa-1 peptide ligands follow the rules of binding that had been established for Qdm [23]. Interestingly, it has been demonstrated for both human HLA-E allotypes as well as their Rhesus macaques orthologs, that the large panel of identified peptides can be presented by all allotypes [35]. This would suggest that the limited polymorphism in these molecules does not greatly affect the peptide ligand repertoire. However, since Qa-1^a^ and Qa-1^b^ have two amino acid differences that affect the P2 anchor pocket, and since it was shown that the P2 anchor residue Met is not conserved in the Qa1 binding peptides, we could envision three different scenarios. Firstly, the peptide ligand pool can slightly differ based on the P2 residue; secondly, the peptide ligand pool is the same for both Qa-1 allotypes but the presentation is slightly altered; or thirdly, the same peptide ligand binds with different affinities, resulting in a preferential association with a single Qa-1 allotype on the antigen presenting cell. Since a large peptide pool has been identified for these non-classical MHC-Ib molecules and since CD8+ T cells specific for Qa-1 and HLA-E exist [35], subtle structural differences in the peptide binding groove of these antigen-presenting molecules could potentially translate into appreciable differences in the selectivity or magnitude of the subsequent T cell response to Qa-1 or HLA-E binding peptides, especially during the cause of altered peptide presentation, such as during tumorigenesis or viral infection.
